# Feasibility of Wire-in-Needle Technique for Ultrasound-Guided Central Venous Catheter Insertion in a Pediatric ICU

**DOI:** 10.7759/cureus.22211

**Published:** 2022-02-14

**Authors:** Ahmad Jorya, Ali Al Shehri, Mohammed Naeem

**Affiliations:** 1 Pediatric Critical Care, Ministry of National Guard Health Affairs, Riyadh, SAU

**Keywords:** ultrasound guided, central venous catheter, pediatric critical care, children, wire-in-needle technique

## Abstract

Objective

For further evaluating the feasibility and safety of wire-in-needle (WIN) technique application for ultrasound-guided central venous catheter (USG-CVC) insertion in a pediatric intensive care unit (PICU).

Methods

We prospectively monitored all patients who underwent central line insertion guided by ultrasound from March 2018 to March 2019. An independent nurse recorded the patient's age, gender, weight and BMI, diagnosis, indication for insertion, blood pressure state, insertion time, line size, number of pricks, and arterial punctures.

Results

A central line was inserted in 141 patients. The author applied the WIN technique in 16 patients, while in 125 patients, the central line was inserted via the traditional technique. The success rate was 100% for the WIN technique arm with zero arterial pricks, and the mean number of needle pricks was 1.1. For the traditional technique arm, the success rate was 90% with three arterial pricks. The mean number of needle pricks was 1.38. The insertion time was 86.25 seconds and 304 seconds for the WIN technique and the standard technique, respectively; this difference was statistically significant (p <0.001).

Conclusion

The WIN technique is feasible and could provide a safe and relatively fast alternative technique for real-time USG-CVC insertion in the PICU. The WIN technique is feasible and not inferior to the standard short-axis technique. A good level of experience related to USG-CVC insertion provides a safe and rapid alternative technique for real-time USG-CVC insertion in the PICU.

## Introduction

Ultrasound-guided central venous catheter (USG-CVC) insertion is a ubiquitous pediatric intensive care unit (PICU) procedure and has received considerable critical attention in recent years. Compared with landmark (LM)-guided techniques, ultrasound use is considered a significant contributing factor to improved CVC insertion success rates, decreased number of cannulation attempts, and reduced incidence of CVC insertion-related complications. Therefore, ultrasound should be utilized for children [1-2].

Confirming the correct guidewire location in the targeted vessel prior to inserting the dilator is of significant importance. Therefore, the American Society of Echocardiography and the Society of Cardiovascular Anesthesiologists recommend that, whenever possible, trained clinicians should use real-time ultrasound during CVC insertion of the internal jugular (IJ) and the femoral vein. This confirms the vascular access and guarantees that the guidewire is absent in the adjacent structures. Additionally, when the short-axis view (SAX) is used solely, extra measurements are recommended to confirm CVC placement, such as manometry with a fluid-filled catheter or transesophageal echocardiographic or fluoroscopic imaging [3].

Different approaches have been used for USG-CVC insertion, including SAX, long-axis view (LAX), and alternative oblique axis approaches. A superior trend has been witnessed for the medial oblique and long-axis plane approach compared to the transverse approach concerning the time of insertion, the number of pricks, and the number of complications, including malpositioning [4-5].

The standard USG-CVC insertion technique is not free of challenges or inadvertent events. For example, the "unseen danger" study by Blaivas M and Adhikari S reported that needle displacement or migration through the posterior vessel wall could occur when the probe is off the skin while the physician reaches the guidewire. Their findings suggested that care must be taken even with USG-CVC placement. The study also recommends considering alternative ultrasound guidance techniques, such as visualization of the vein and needle in the longitudinal axis [6]. Another study described a common problem preventing the introduction of the guidewire into the IJ vein, which stems from the frequent displacement of the introduced needle that takes place during detachment of the syringe or the advancement of the guidewire over the needle [7].

CVC insertion could be more challenging in the pediatric population given the smaller vessel diameter and, on many occasions, the lack of blood backflow even if the needle is in place.

The WIN technique, which was first described in 2013 [7], may assist overcome the previous challenges. A paucity of literature concerning this technique has been found in pediatric and adult patients, of which all the patients were hemodynamically stable and well prepared for elective procedures. Additionally, the operators were surgeons or anesthesiologists who cannulated the IJ vein [8-10].

This paper aims to evaluate the feasibility of the WIN technique application for sick children admitted to a PICU and compare its efficacy with the standard technique traditionally used for USG-CVC insertion. This is the first study evaluating the feasibility of the WIN technique in pediatric critical care units to the best of our knowledge.

## Materials and methods

Technique

**Figure 1:**
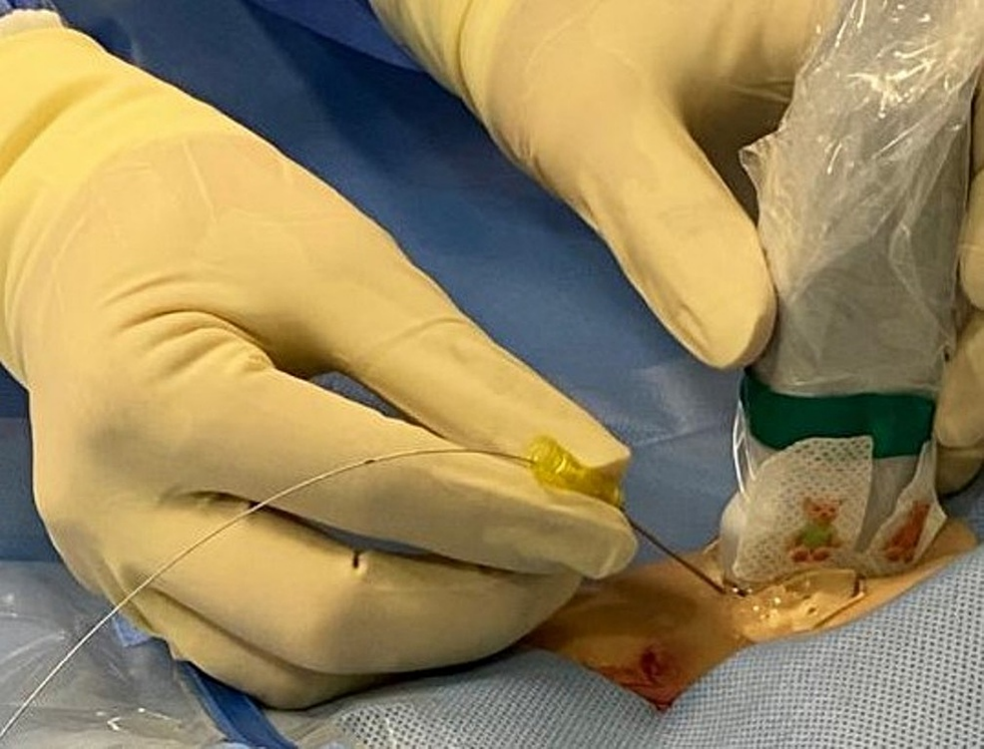
Puncturing the skin parallel to the linear ultrasound probe with a guide wire-loaded needle.

Following the standard aseptic technique before CVC insertion, the operator scans the targeted area with a linear ultrasound probe along the longitudinal axis, relying on the ultrasonic characteristics to differentiate the vein from the adjacent artery. After determining the vein, the operator loads the needle supplied in the CVC kit with the guidewire (Figure 1).

Then, while utilizing continuous real-time US imaging, the operator punctures the skin parallel to the probe axis and observes the guidewire-loaded needle advancement by ultrasound until it is visualized in the targeted vein.

**Figure 2:**
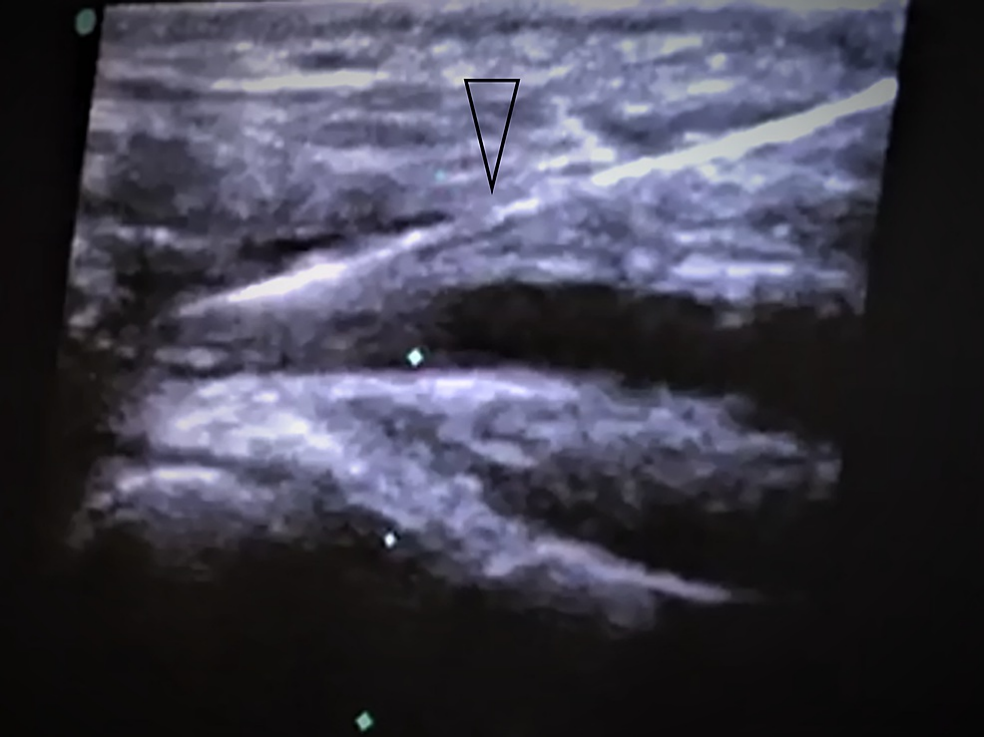
Guidewire-loaded needle in the vein. Triangle pointed at the guidewire-loaded needle.

The operator, immediately without taking off the probe, advances the guidewire into the vein and continues observing its ultrasonographic brightness inside the vein.

**Figure 3:**
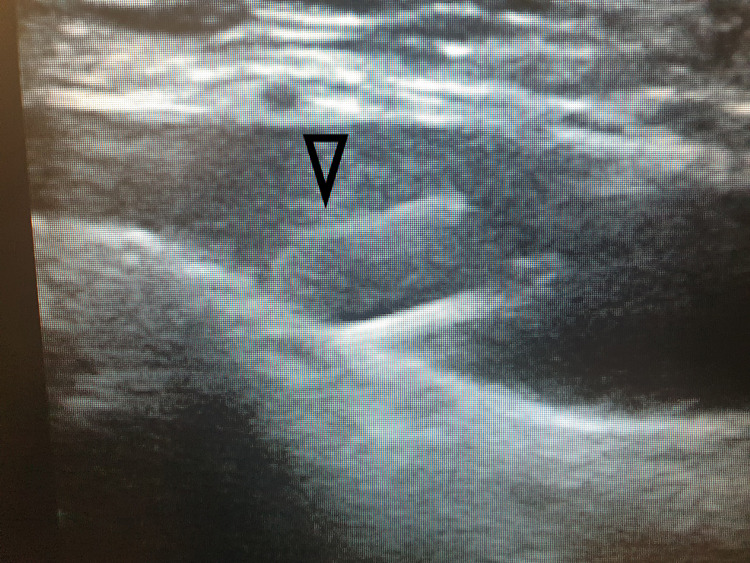
Guidewire in the vein after removing the needle. Arrow pointed at the guidewire within the vein after needle removal.

At this point, the operator uses his dominant hand to advance the guidewire without assistance. At the same time, the needle is fully visualized in the targeted vein on the US screen to eliminate the risk of needle displacement. Following this confirmation, the needle will be removed, and a dilator will be inserted over the lifted guidewire. At the final stage, the CVC is advanced to its final position. The operator confirms accurate catheter placement again with the US before announcing the end of the procedure. Video 1 demonstrates the whole process.

**Video 1:** Wire-in-needle technique for central venous catheter insertion in pediatrics.

Methods

This prospective feasibility study was conducted in a tertiary hospital's PICU from March 2018 to March 2019. All patients aged between 1 day and 14 years who underwent CVC insertion were eligible for inclusion. Consent for CVC insertion was obtained upon admission to the PICU by the admitting physician. The study was reviewed and approved by King Abdullah International Medical Research Center. The participant's legal guardian/next of kin provided written informed consent to participate in this study.

Inclusion criteria

All patients admitted to PICU and required CVC insertion were eligible. Ten intensivists, with vast experience ranging from 2 to 8 years in PICU, performed the procedures. Nine out of ten practice the standard short-axis out-of-plane technique. The first author, a qualified pediatric intensivist with five years of experience, performed the procedure via the longitudinal in-plane access for both femoral and IJ veins (a hokey-stake probe used for the small children who needed IJ-CVC insertion). The rest of the operators strictly implied the short-axis out-of-plane technique for both IJ and femoral vein cannulation.

Due to the emergency nature of the procedure that merits prompt insertion of CVC to the sick child and the work dynamics in PICU, patients allocation and the choice of the insertion technique were at the discretion of the operator caring for the patient.

Statistical analysis

Descriptive data for all demographic and anthropometric variables related to PICU admission were generated, including patient ID, age, sex, BMI indication for CVC insertion, blood pressure before insertion, insertion time, anatomical site, size of the catheter, number of skin pricks, and any accidental arterial pricks. The insertion time was defined as the time interval between the first skin prick until the guidewire was confirmed inside the targeted vein. Management and analysis were performed using Microsoft Excel 2007 and SPSS software version 26. Categorical data were expressed as percentages, and continuous data were expressed as means. The groups were compared using Fisher's exact test, and significance was expressed as a p-value (less than 0.05). Primary outcomes were the CVC insertion success rate and inadvertent complications such as arterial pricks. Secondary outcomes were the insertion time and the number of skin pricks.

## Results

The total number of admissions to the unit during the study was 810 patients. Ultrasound was used to obtain CVC access for 141 patients. The first author performed the WIN technique on 16 patients, while the other nine operators performed 125 USG-CVC insertions via standard techniques. The patient demographic and hemodynamic characteristics are shown in Table 1.

**Table 1:** Demographic and hemodynamic characteristics. *Categorical data presented as number (percentage). **Continuous data presented as mean (SD).

	WIN technique (n=16)	Standared technique (n=125)		p-value
Male (percentage)*	7 (43%)	67 (53%)		0.4526
Mean age (mo)**	49 (54)	39 (71)		0.588
Mean weight (kg)**	13.6 (54)	13.9 (61)		0.974
Mean BMI (kg/m2)**	15.2 (5.4)	15.3 (4.7)		0.937
Femoral*	13 (81%)	74 (59.2%)		0.09
Internal jugular*	3 (18%)		51 (40.8%)	0.076
Hypotension*	6 (37.5%)		43 (43.4%)	0.649

There was no statistically significant difference between the two groups regarding the demographic data (p > 0.05). Procedure-related data are shown in Table 2.

**Table 2:** Comparison of the syringe-free technique and standard technique. *Categorical data presented as number (percentage). **Continuous data presented as mean (SD).

	WIN technique (n=16)	Standared technique (n=125)	p-value
Mean Cannulation time** (sec)	86 (73)	304 (432)	<0.001
Mean number of pricks	1.1 (0.54)	1.4 (0.8)	0.927
Success rate (%)*	100	90	0.187
Complication (Unintended arterial puncture)	0	3	0.484

The mean insertion time was 86 seconds and 304 seconds for the WIN and standard techniques, respectively; this difference was statistically significant (p <0.001). The mean number of needle pricks was 1.1 and 1.38 for the WIN technique and standard technique, respectively, and this difference was not significant. The success rate was 100% with zero arterial pricks in the WIN technique arm versus a success rate of 90% and three arterial pricks found in the standard technique arm.

All USG-CVC insertions were either in the femoral vein or IJ vein. Among the groups, the ratios of CVC insertions in the femur and IJ were statistically insignificant (p-values: 0.09 and 0.076, respectively). In addition, no significant difference was found in hemodynamic instability prevalence between the two groups (37.5% versus 43.4%, p-value: 0.649).

Subgroup analysis

We compared the time of insertion among patients who were cannulated in the femoral vein from both arms. The statistical significance of time insertion difference has not been altered by the site of insertion 79 vs. 425 seconds (p-value <0.001) in WIN technique arm and standard technique, respectively (Table 3).

**Table 3:** Sub-group analysis for the time of insertion difference in relation to the site of insertion.

	WIN technique: No. of patients	WIN technique: Average time (sec)	Standard technique: No. of patients	Standard technique: Average time (sec)	P-value
Femoral vein	13	79 sec	44	425 sec	<0.001

Linear and variable logistic regression has been used to further analyze the two arms in the presence of unequal sample size. The multiple linear of the factors affecting cannulation time (seconds) is demonstrated in Table 4.

**Table 4:** Multiple linear regression of the factors affecting cannulation time (seconds). Dependent variable: Time (seconds).

	Parameter	P-value	B	95% CI for B	
				Lower	Upper
Intercept		<0.001	390.7	228.5	552.8
Age		0.117	-2.4	-5.3	0.6
Weight		0.529	4.4	-9.4	18.3
Technique	WIN	0.034	-252.6	-485.5	-19.7
	Standard (ref)		0.0		
Sex	Female	0.207	-93.6	-239.8	52.6
	Male (ref)		0.0		
Site	Femoral	0.357	69.7	-79.3	218.7
	Jugular (ref)		0.0		

The WIN technique had significantly shorter cannulation time than the standard technique, (B = -252; 95% CI = [-485.5 to -19.7]; p-value = 0.034). The multiple logistic regression of factors affecting procedure success rates is presented in Table 5.

**Table 5:** Multiple logistic regression of factors affecting procedure success rate. Dependent variable: Status (success).

	Parameter	P-value	AOR	95% CI for AOR	
				Lower	Upper
Weight		0.287	1.11	0.92	1.35
Age		0.968	1.00	0.97	1.04
Technique	WIN	0.552	1.98	0.21	18.73
	Standard (ref)		1.00		
Sex	Female	0.64	0.75	0.22	2.55
	Male (ref)		1.00		
Site	Femoral	0.129	0.34	0.08	1.37
	Jugular (ref)		1.00		

Although there was a higher chance of success when using the WIN technique than the standard, there was no significant difference (AOR = 1.98; 95% CI = [0.21 to 18.73]; p-value = 0.552).

## Discussion

This study set out to evaluate the feasibility and safety of the WIN technique for CVC insertion in critically ill children. Very little was found in the literature on the feasibility and safety of this technique in pediatric critical care unit settings.

A study by Sallam et al conducted in 2018 described this technique for pediatric patients with cancer who needed Port-A-Cath insertion. They reported a success rate of 100%, with almost one skin prick per patient. These findings are consistent with our study's findings; however, the operator was a surgeon, and the procedure was performed for stable children under general anesthesia in the operating theater. The insertion site was the IJ vein, which was accessed using an Angiocath set (not a CVC kit) using the short-axis in-plane technique. The operator required assistance to advance the micro guidewire and another step to rewire the dilator with a CVC set guidewire [9].

A study by Matias F in 2015 described the syringe-free WIN technique in adult patients The mean time for insertion was 43.9 seconds, and a mean of 1.2 pricks per patient was made, matching our study's results. In contrast to our study, an anesthesiologist performed the procedure, and the patients had been well prepared preoperatively. The site of insertion was the IJ vein [10].

In 2018, Ince I et al. conducted a prospective, randomized study in adults that compared the syringe-free technique with a short-axis out-of-plane technique. Although the insertion site was the IJ vein and the patients were hemodynamically stable with normal blood pressure, interestingly but not surprisingly, similar results were in line with ours. The mean time for insertion was 43.9 seconds, and a mean of 1.2 pricks per patient was found. Those studies recommended evaluating this technique in the pediatric population where the vessels are small, and the patients demonstrate hemodynamic instability signs [11].

Unlike previous studies, our study included unstable children in a critical care unit. In addition, the operator was a pediatric intensivist, and the operator completed successfully the WIN technique without assistance.

Our findings, consistent with a current prospective randomized control study, compared the WIN technique with short-axis out-of-plane standard technique. Our results corroborate the findings of a great deal of that found in this study, where they noticed that the time of insertion was significantly shorter in the WIN arm. In contrast, no other differences between the two arms were present. This supports the conclusion that the WIN technique is feasible and safe and may provide an alternative approach for CVC insertion in PICU [12].

Our study revealed exciting findings in the settings of critically ill pediatric populations. Neither the presence of hypotension (37.5%) nor cannulation of the femoral vein (81%), which is far smaller in diameter than the IJ vein, affected the success rate (100%) or provoked inadvertent arterial pricks (zero). In addition, our study demonstrates a comparative time for insertion and number of pricks with those found in similar studies.

Although our study lightened the feasibility and safety of this technique in the PICU population, it has many limitations. First, it is a single-center study performed by a well-trained operator. Second, the WIN technique was performed in a limited number of patients, which does not reflect the variety in disease types and severity of patients admitted to the critical care unit. Third, the lack of randomization and the small number of patients in the WIN technique arm is another limitation in our study. This is attributed to the work dynamics in our busy unit. We have ten intensivists, nine of them practice the short-axis out-of-plane technique, and most of the procedures occur overnight for the new admissions. This mandates that the attending intensivist carry out the procedure according to his/her preference, which makes the randomization very difficult because the WIN technique is performed solely by the first author, who may not be on-site to perform the procedure if the patient is allocated to the WIN technique arm. Finally, this technique is considered operator-dependent because, for nonexpert US users, there is a risk of accidental arterial pricking due to the inability to differentiate the vein from the adjacent artery. In this case, a minor deviation of the probe during the procedure can lead to direct insertion of the needle into the misrecognized artery. Therefore, a good level of experience and training is required before embracing this technique.

## Conclusions

The WIN technique is a relatively newer add-on skill in USG-CVC insertion procedures. In the setting of pediatric intensive care population, our study shows that this technique is feasible and could provide a safe and relatively fast alternative approach for real-time USG-CVC insertion. With an appropriate level of experience, one operator can perform the procedure safely, effectively, and fast enough without assistance. Further studies on larger populations and different operators are needed to assess this technique in critically ill children.
